# MCR Expression Conferring Varied Fitness Costs on Host Bacteria and Affecting Bacteria Virulence

**DOI:** 10.3390/antibiotics10070872

**Published:** 2021-07-19

**Authors:** Wan Li, Zhihai Liu, Wenjuan Yin, Lu Yang, Lu Qiao, Shikai Song, Zhuoren Ling, Ruicheng Zheng, Congming Wu, Yang Wang, Jianzhong Shen

**Affiliations:** 1 Department of Veterinary Pharmacology and Toxicology, College of Veterinary Medicine, China Agricultural University, Yuanmingyuan West Road No. 2, Beijing 100193, China; wanli20173050431@cau.edu.cn (W.L.); yanglu2016@cau.edu.cn (L.Y.); qiaoatshandong@163.com (L.Q.); songshikai@cau.edu.cn (S.S.); lingzrformal@163.com (Z.L.); b20203050400@cau.edu.cn (R.Z.); wucm@cau.edu.cn (C.W.); wangyang@cau.edu.cn (Y.W.); 2 College of Chemistry and Pharmaceutical Sciences, Qingdao Agricultural University, Qingdao 266109, China; lzh@qau.edu.cn; 3 Department of Microbiology and Immunology, College of Husbandry and Veterinary Medicine, Henan Agricultural University, Zhengzhou 450046, China; 4 Key Laboratory of Pathogenesis Mechanism and Control of Inflammatory-Autoimmune Diseases of Hebei Province, College of Basic Medical Science, Hebei University, Baoding 071002, China; ywj0501@hbu.edu.cn

**Keywords:** *mcr-1~5*, fitness cost, virulence, prevalence

## Abstract

Since the first report of the plasmid-mediated, colistin-resistant gene, *mcr-1*, nine *mcr* genes and their subvariants have been identified. The spreading scope of *mcr-1~10* varies greatly, suggesting that *mcr-1~10* may have different evolutionary advantages. Depending on MCR family phylogeny, *mcr-6* is highly similar to *mcr-1* and *-2*, and *mcr-7~10* are highly similar to *mcr-3* and *-4*. We compared the expression effects of MCR-1~5 on bacteria of common physiological background. The MCR-1-expressing strain showed better growth than did MCR-2~5-expressing strains in the presence of colistin. LIVE/DEAD staining analysis revealed that MCR-3~5 expression exerted more severe fitness burdens on bacteria than did MCR-1 and -2. Bacteria expressing MCRs except MCR-2 showed enhanced virulence with increased epithelial penetration ability determined by trans-well model (*p* < 0.05). Enhanced virulence was also observed in the *Galleria mellonella* model, which may have resulted from bacterial membrane damage and different levels of lipopolysaccharide (LPS) release due to MCR expression. Collectively, MCR-1-expressing strain showed the best survival advantage of MCR-1~5-expressing strains, which may partly explain the worldwide distribution of *mcr-1*. Our results suggested that MCR expression may cause increased bacterial virulence, which is alarming, and further attention will be needed to focus on the control of infectious diseases caused by *mcr*-carrying pathogens.

## 1. Introduction

Colistin is considered a “last-resort” antibiotic for treating infections caused by multidrug-resistant bacteria. However, the therapeutic effect of colistin has been greatly compromised by the emergence of the mobile colistin-resistance gene *mcr-1* [1]. *mcr-1*-positive bacteria have been detected worldwide from humans, animals, the environment, and aquatic sources [2,3,4,5]. The *mcr* gene family has expanded to different bacterial species, such as *Escherichia coli* in Belgium (*mcr-2*) [4], *Enterobacteriaceae* and *Aeromonas* in China (*mcr-3*) [5], *Salmonella* and *E. coli* in Spain and Belgium (*mcr-4*) [6], *Salmonella* in Germany (*mcr-5*) [7], *Moraxella* spp. in the United Kingdom (*mcr-6*) [8], *K. pneumoniae* in China (*mcr-7, -8*) [7,9], *Salmonella enterica* serotype *Typhimurium* in the USA (*mcr-9*) [10], and *Enterobacter roggenkampii* in China (*mcr-10*) [9]. *mcr* genes have a large and diverse epidemiological distribution, while *mcr-1*-positive bacteria have been reported across six continents. However, the distribution of the other *mcr* genes (*mcr-2~10*) is relatively limited [11]. Whether the fitness costs for bacteria caused by *mcr* genes can influence the gene’s dissemination remains uncertain.

The biological costs imposed by a plasmid-mediated resistance gene affect its maintenance in the bacterial community, because resistant strains with the extra burden could easily be replaced by susceptible strains in the absence of antibiotic pressure [12,13]. Previous studies have verified that *mcr* gene expression affected bacterial growth conditions [12]. Expression of MCR-1 and MCR-3 exerted fitness costs on the first 50 generations of host bacteria, and compensatory mutations can alleviate these costs [12]. *mcr*-carrying plasmids with different replicon types are varied in their fitness burdens on host bacteria. The IncI2, IncHI2, and IncX4 plasmid-carrying *mcr-1* genes are stable and hardly affect bacterial growth, which explains why most reported *mcr-1*-bearing plasmids belong primarily to these types [12,13]. Although the fitness costs of *mcr-1*-carrying strains have been thoroughly studied under different conditions, few studies have compared the fitness costs of different *mcr* genes in a common physiological background.

MCR transfers the phosphoethanolamine (pEtN) on lipid A, alters its negative charge, and decreases the colistin-binding efficiency. However, MCR-mediated lipid A modification and its subsequent effect on bacterial virulence remain largely unexplored. It is reported that there is a complicated relationship between colistin resistance and bacterial virulence [14]. Increased colistin resistance along with decreased virulence was observed in *E. coli* [1]. However, some *A. baumannii* exhibited increased colistin resistance but also increased virulence gene expression [15]. Worryingly, some pathogenic clonal lineages, such as *E. coli* ST131, have been isolated from clinical patients, food animals, and environments harboring *mcr*, which is alarming because carrying of *mcr* in this clonal may not only result in therapeutic failure, but also varied virulence to host animal [16,17,18]. All this indicates that it is necessary to figure out the association between MCR mediated colistin resistance and virulence variation in bacteria.

Herein, in this study, we analyzed the fitness costs and virulence of bacteria carrying *mcr-1~5*, which contributed to exploring the causes of *mcr* genes’ widespread distribution and was a reminder of the risk of *mcr*-carrying bacteria outbreak.

## 2. Materials and Methods

### 2.1. Bacterial Strains, Plasmids, Cell Lines, and Culture Conditions

Bacterial strains, plasmids, and cell lines used in this study are listed in Table 1. Bacteria carrying *mcr-1~5* were isolated previously and kept in our lab. Strains were grown in LB broth (Luqiao, Beijing, China) under 37 °C with proper antibiotics. *E. coli* TOP10 (*mcr-1~5*/pBAD) was constructed by cloning *mcr-1~5* genes in frame into arabinose-inducible pBAD-hisA expression vector with primers listed in Table A1; hence, the expression of MCR-1~5 can be regulated with L-arabinose (Sigma-Aldrich, St. Louis, MO, USA) induction. *E. coli* TOP10 transformed with empty pBAD vector was used as the control group. Bacteria were cultured in Luria–Bertani (LB) medium (Luqiao, Beijing, China) at 37 °C. The growth medium was supplemented with antibiotic and/or L-arabinose.

Cell line Caco-2 was grown in 12 well trans-well cell culture inserts (Nunc, Rochester, NJ, USA) to confluency. The inserts were cultured 18–22 days at 37 °C, 10% CO_2_ in minimum essential medium (MEM, Gibco, Waltham, MA, USA) supplemented with fetal bovine serum (FBS, Gibco, Waltham, MA, USA), 1% non-essential amino acids (Gibco, Waltham, MA, USA), and 1% penicillin/streptomycin (Gibco, Waltham, MA, USA). The cell culture medium was changed every other day until the cells were fully differentiated.

### 2.2. Antimicrobial Susceptibility Testing

Colistin susceptibility of constructed strains was assessed by broth microdilution according to the European Committee on Antimicrobial Susceptibility Testing (EUCAST). Briefly, cells were resuspended to 0.5 McFarland standard and used to inoculate 96-well microtiter plates (Corning Inc., Corning, NY, USA) containing cation-adjusted Mueller–Hinton medium (Luqiao, Beijing, China) with colistin (0.06~128 μg/mL). L-arabinose was added to experimental wells to a final concentration of 0~0.2% (*w/v*). Plates were incubated 16~18 h at 37 °C. Experiments were carried out in duplicate.

### 2.3. Bacteria Growth Kinetics Detection

Bacteria were streaked on LB agar supplemented with ampicillin. Single clones were inoculated into 1 mL of LB broth supplemented with 100 mg/L ampicillin and shaken at 37 °C for 6 h to log phase. The bacteria were diluted to a turbidity of 0.5, which was diluted 100 times in LB broth with L-arabinose 0.02% (*w/v*). Bacteria only or bacteria as well as drugs were added to the 96-well plate then placed in a microplate reader and cultured at 37 °C. The growth state of the bacteria was monitored for 24 h, and the OD value of each well was measured intermittently using a wavelength of 600 nm. The growth curves of the bacteria were fitted using GraphPad prism by triplicate growth kinetics assays.

### 2.4. Confocal Microscopy Imaging Using LIVE/DEAD Staining 

Strains were grown overnight in LB broth supplemented with 100 mg/L ampicillin at 37 °C (120 rpm). Overnight cultures were standardized to OD600 0.05 and inoculated (1:10; *v/v*) into fresh LB broth for 2 h (37 °C; 120 rpm). The L-arabinose (0.02%, *w/v*) was added and further incubated for 2 h. The strains were stained with 6% LIVE/DEAD^®^ (*v/v*; BacLightTM Bacterial Viability Kit, Invitrogen, CA, USA) in phosphate-buffered saline before CLSM imaging (Leica TCS SP8, Leica, Wetzlar, Germany) with a ×63 lens. The CLSM images were analyzed using ImageJ version 1.52a analysis software.

### 2.5. Membrane Permeability Test Assay

Strains were inoculated in LB broth with 0.02% L-arabinose and cultured at 37 °C, 180 rpm for 2 h. NPN (1-N-phenylnaphthylamine, Sigma-Aldrich, St. Louis, MO, USA) with final concentration at 10 μM was added and incubated at 37 °C for 30 min in the dark; fluorescence was detected at the excitation and emission wavelengths of 350 and 420 nm, respectively.

### 2.6. Cell Infection Experiment

To detect bacterial ability to penetrate the epithelial barrier, we challenged the apical surface of Caco-2 monolayers by the addition of 1 × 10^6^ CFU/mL strains with or without *mcr* genes after L-arabinose induction. After incubation at 37 °C, the lower cell liquid was gathered and inoculated on LB agar. After overnight incubation, CFU on agar was counted. Every time before and after the experiment, *mcr* genes were detected by PCR with primers pBAD-F (5′ATGCCATAGCATTTTTATCC3′) and pBAD-R (5′GATTTAATCTGTATCAGG3′) to make sure the gene is still maintained in host bacteria.

### 2.7. G. mellonella Infection Model

In vivo virulence of strains was evaluated using *G. mellonella* infection model, as described by Qiue [1]. Briefly, bacterial pellets were washed and diluted to an appropriate density with PBS. We injected 10 μL aliquots of serially diluted bacterial suspension (10^8^ CFU/mL) into the hemocoel of each larvae through the rear left proleg. A group of 5 larvae was randomly chosen to inject inoculation in triplicate. After injection, larvae were incubated at 37 °C, and the melanization of larvae were observed 1 h after infection. In all cases, no dead larvae were observed in the control groups.

After *G. mellonella* was ground, the homogenate was diluted in gradient and the bacteria was removed by filtration with 0.2 μm filters. The samples were quantified for the presence of LPS using the LAL. Briefly, samples and LPS standards (Sigma-Aldrich, St. Louis, MO, USA) were diluted to the concentration between 0.1 and 1 endotoxin unit and incubated for 10 min with LAL lysates (Thermo Scientific™, Waltham, MA, USA) at 37 °C. Then, the samples with LAL lysates were treated with chromogenic reagent and color stabilizer reagents. LPS levels were determined by detecting absorptions at 405 nm.

### 2.8. Statistical Analysis

All assays were repeated three times. Data are expressed as the mean ± standard error of the mean (SEM). Statistical significance (***, *p* < 0.001; **, *p* < 0.01; *, *p* < 0.05; ns (not significant)) was calculated via two tailed *t*-test to compare treatment/experimental and control groups. Analyses was performed by using GraphPad prism 8.0 (GraphPad Software, La Jolla, CA, USA).

## 3. Results

### 3.1. Mcr-1~5 Mediated Colistin Resistance Levels

**Figure 1:**
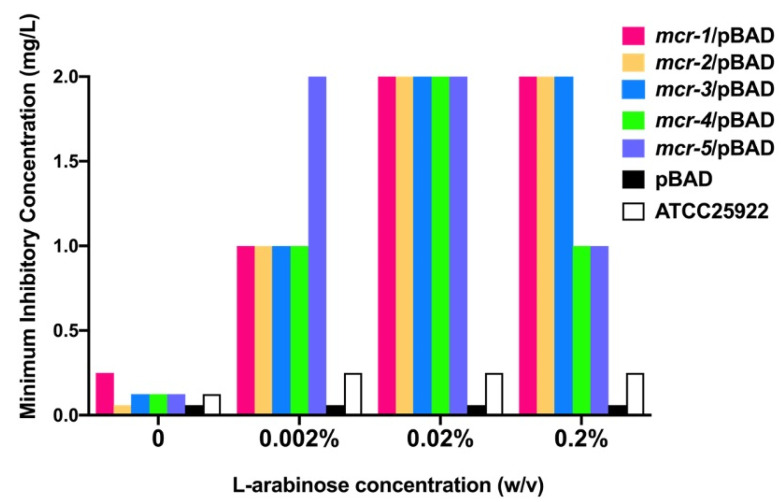
MICs of colistin to constructed strains in the presence of 0–0.2% (*w/v*) L-arabinose in broth.

To compare *mcr*-mediated resistance to colistin in a common physiological background, we expressed MCR in TOP10 *E. coli* by cloning *mcr* into pBAD expression vectors, which was confirmed by PCR and sequencing (Table 1). MCR expression was induced under different L-arabinose concentrations after 2 h of inoculation in LB broth. As the concentration of L-arabinose increased within 0~0.02% (*w/v*), the minimum inhibitory concentrations (MICs) against colistin increased (Figure 1). However, when the L-arabinose concentration reached 0.2%, the MICs for colistin of the *mcr-1~3*-carrying strains were the same as those at 0.02%, while those of *mcr-4* and *-5* were decreased by half (1 mg/L). All constructed strains exhibited maximal MICs (2 mg/L) to colistin under 0.02% L-arabinose, which was 8~32 times higher than the MICs without L-arabinose induction, and no higher MICs were observed. Therefore, we used 0.02% L-arabinose as the induction concentration in subsequent experiments.

### 3.2. MCR-1~5 Expression Conferred Varied Fitness Costs on the Host Strain

**Figure 2:**
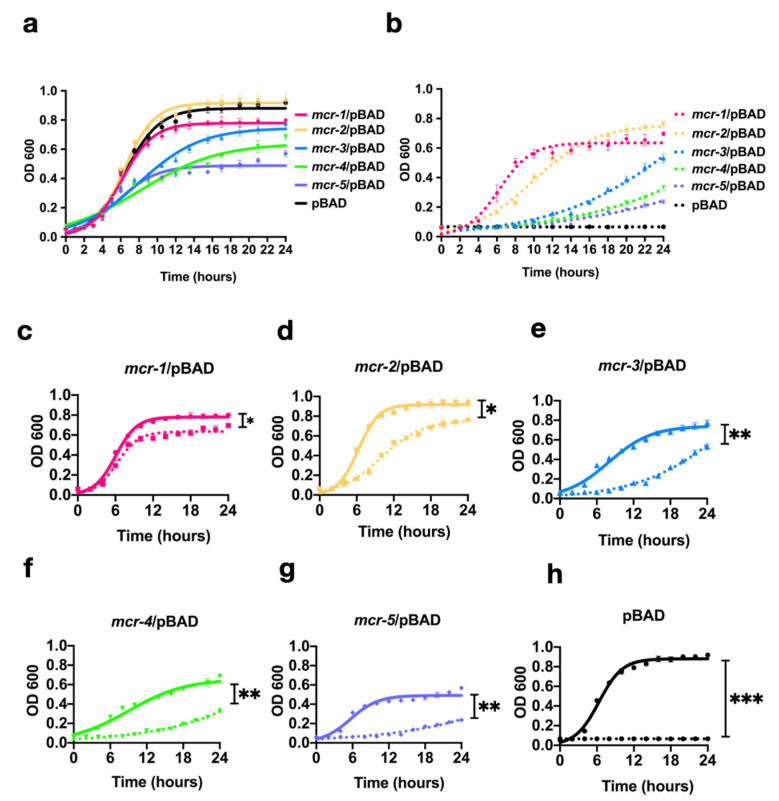
Growth kinetics of MCR-expressing strains in the presence or absence of 0.5 mg/L colistin in broth. (**a**) Growth kinetics of bacteria without colistin and (**b**) with 1 mg/L colistin added to the medium. (**c**–**h**) Comparisons of each MCR-expressing strain with or without colistin added to the medium. Solid lines represent the absence of colistin; dashed lines represent addition of colistin to the medium. * *p* < 0.05, ** *p* < 0.01, *** *p* < 0.001.

To determine the impact of MCR-1~5 expression on bacterial growth, we constructed growth curves in the presence/absence of 1 mg/L colistin. All data were fitted with logistic regression to show the host strain growth phase. The bacterial growths of MCR-3, -4, and -5 overexpression without colistin were significantly inhibited in the log phase (*p* < 0.01) compared with that of the control group, while MCR-1 imposed a slight fitness disadvantage in the lag phase (*p* < 0.05; Figure 2). Surprisingly, MCR-2 showed a similar growth kinetic to that of the host strain (Figure 2a).

MCR-1- and MCR-2-expressing strains could grow with different kinetics in the log phase with 1 mg/L colistin. However, the growths of strains expressing MCR-3, -4, and -5 were significantly inhibited in the log phase (*p* < 0.01), and no normal growth curves could be detected after 24 h (Figure 2c,g). Interestingly, when colistin was added, MCR-2-expressing strains showed poorer growth than did MCR-1-expressing strains (Figure 2c,d) in the logarithmic phase.

**Figure 3:**
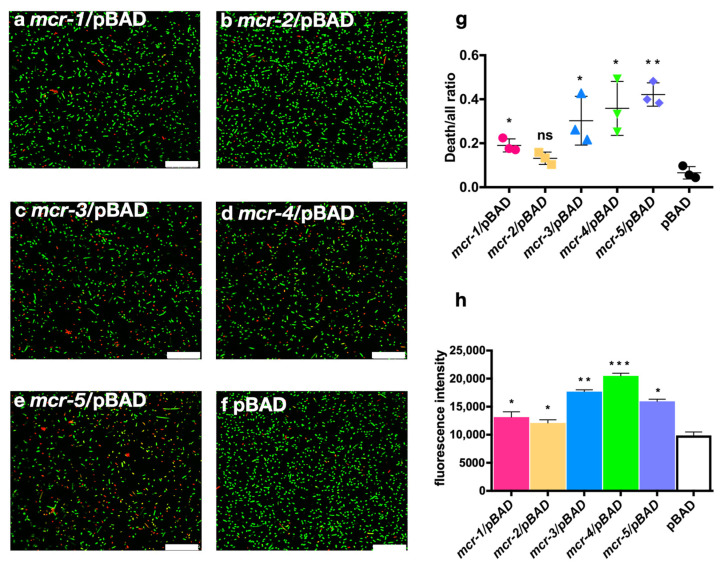
LIVE/DEAD staining of strains expressing MCR genes. (**a**–**f**) Confocal observation of strains expressing MCR-1–5 with 0.02% L-arabinose induction. (**g**) Statistical analysis of LIVE/DEAD staining images; images were analyzed using ImageJ software. (**h**) Membrane permeability of strains expressing MCR genes. Fluorescence intensity of NPN after 5 min of incubation with strains was detected at 420 nm. * *p* < 0.05, ** *p* < 0.01, *** *p* < 0.001, ns: no significant difference. Scale bars represent 25 μm.

We performed LIVE/DEAD double-staining assays to reflect cell viability (Figure 3a–f). Compared with the control bacteria that only harbored pBAD, MCR-1, -3, -4, and -5 expressions caused significantly high ratios of dead bacteria to all bacteria (*p* < 0.05), while MCR-2 expression yielded comparable ratios to those of the control group (Figure 3g).

Because 1-N-phenylnaphthylamine (NPN) is hydrophobic, it cannot penetrate intact membranes, whereas NPN uptake may be enhanced in bacteria with damaged outer membranes. We detected the outer membrane permeability of the strains after L-arabinose induction by measuring the NPN uptake in bacteria. The strains expressing MCR-1~5 showed significantly increased membrane permeability (*p* < 0.05), with the order of membrane damage being MCR-4 > MCR-3 > MCR-5 > MCR-1 > MCR-2. Membrane permeability in the MCR-2-expressing strains was similar to that of the control bacteria (Figure 3h).

### 3.3. MCR Expression Increased Bacterial Virulence In Vitro and In Vivo

**Figure 4:**
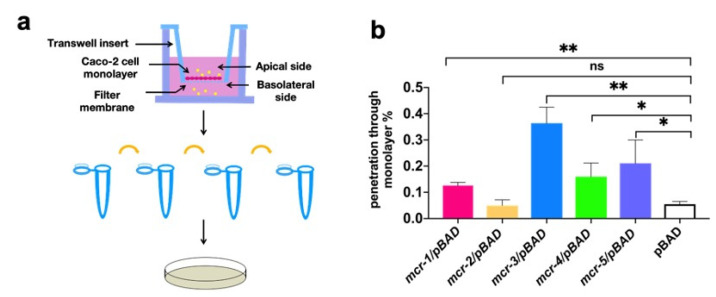
Bacterial virulence evaluated using trans-well assays and *G. mellonella*. (**a**) Study design of the trans-well assay. (**b**) Percentage of MCR-expressing strains that passed through the Caco-2 cell monolayer at 2 h measured by viable counting. * *p* < 0.05, ** *p* < 0.01, ns: not significant.

Intestinal epithelial cells can generate various types of barriers to protect the intestinal mucosa from commensal and pathogenic microorganisms. Here, we explored whether MCR-catalyzed lipid A modification could affect the ability of bacteria to penetrate the epithelial barrier via a trans-well model in vitro (Figure 4a). The translocated bacteria were quantified by counting the viable cells. Bacteria expressing MCR-1, -3, -4, and -5 showed a significantly higher capacity to translocate the Caco-2 monolayer than did the control group (*p* < 0.05), where MCR-3-expressing strains showed the highest capacity to penetrate the epithelial barrier. However, MCR-2-expressing strains showed a similar penetration capacity to the non-pathogenic pBAD control group (Figure 4b). MCR-1 expressing strain showed intermediate ability to penetrate Caco-2 monolayer, which is higher than MCR-2 but lower than MCR-3~5-expressing strains.

**Figure 5:**
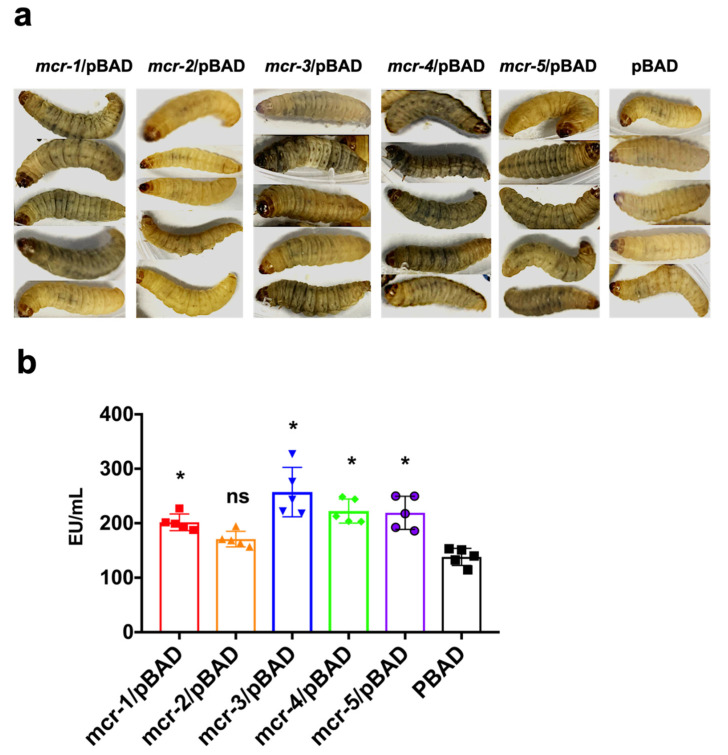
Bacterial virulence evaluated in a *G. mellonella* model. (**a**) Melanization of *G. mellonella* infected with MCR-expressing strains. (**b**) LPS concentrations in *G. mellonella* after infection. * *p* < 0.05, ns: not significant.

We used *G. mellonella* to evaluate bacterial virulence in vivo. The movement abilities of *G. mellonella* infected with strains expressing MCR-1, -3, -4, and -5 were shown to be rapidly decreased, while the skin of the larvae quickly darkened within 1 h after injection (Figure 5a). In this model, MCR-2-expressing strains still showed similar results to those of the control group.

We detected the LPS concentrations in the fluid of ground *G. mellonella*. The *G. mellonella* infected with bacterial strains expressing MCR-1, -3, -4, and -5 contained significantly higher LPS concentrations in the larvae grinding fluid (*p* < 0.05) than did the MCR-2-expressing strains, which were similar to those of the control group injected with only pBAD-harboring strains (Figure 5b).

## 4. Discussion

MCR family proteins catalyze an additional pEtN group on lipid A, which results in attenuating the bacterium’s affinity for colistin, and obviously compromises the efficacy of colistin as a last-resort antibiotic. Although *mcr* genes (*mcr-1~5*) belong to one family, their prevalence is significantly varied. Of *mcr-1~5*, *mcr-1* is the most widely disseminated variant, however, there are only sporadic reports of *mcr-2~5* which dissemination across a limited area [11]. Resistance-associated fitness costs may inhibit gene further spread in the absence of antibiotics. In our research, the expressions of MCR-3~5 conferred more severe fitness burdens on bacteria than those of MCR-1 and -2, suggesting that *mcr-1* and *-2* exhibit better mutual adaptation with the host bacteria (in our study is *E. coli*) than do *mcr-3~5*. On this basis, *mcr-1* and *-2* should have a similar prevalence level; however, *mcr-1* is much more widespread than *mcr-2*. We further compared the growths of strains under colistin exposure; on this occasion, MCR-1-expressing strains showed the best growth among MCR-1~5, which might explain the widespread of *mcr-1* but not *mcr-2*. Currently, most of the *mcr-1*-positive isolates are limited to *Enterobacteriaceae*. A global surveillance revealed that, among the 29 *mcr*-positive *Enterobacteriaceae* isolated from 2014 to 2016, 24 (82.75%) carried *mcr-1* or its variants [21]. A report on *mcr-1~5* prevalence in *Enterobacteriaceae* showed that 54 of 61 *mcr*-positive isolates were *E. coli* [22]. Colistin-resistant *E. coli* isolates in food-producing animals in Italy during 2014–2015 were shown to carry *mcr* genes; among the 15 *mcr*-positive *E. coli*, 14 harbored *mcr-1*, and only one tested positive for *mcr-4* [23]. The better mutual adaptation between *mcr-1* and *E. coli* in terms of our result of varied fitness cost may partially explain why *mcr-1* is the most prevalent gene in the *mcr* family. Inconsistent results about fitness cost conferred by MCR expression have been reported in *K. pneumoniae* and *E. coli*, with reduced biological fitness being observed [1,24]. However, *mcr-1* was also reported as being able to confer fitness advantages to the host when carried by IncI2 and IncX4 plasmids, suggested different host strain and plasmid combinations may exert varied fitness effects. This might explain why IncI2 and IncX4 are the major vehicles of *mcr-1* worldwide [15], as certain genes on these plasmids may help strains compensate for the fitness costs of MCR expression [25]. Except for fitness cost, the types of plasmids that carry *mcr* genes could also result in large different in its distribution, as the globally distributed *mcr-1* has been associated with the broad host range plasmids including IncI2, IncX4, and IncHI2 [13,26]; however, *mcr-2* was currently only observed carried by IncX4 plasmid [4,6]. Whether there are other contributing factors for the widespread of *mcr-1* will be explored in subsequent studies.

Acyl chains and phosphate groups of disaccharides are the major moieties that contribute to endotoxicity of lipid A [27]. Removing of phosphate groups from lipid A makes some bacterial species less toxic [28]. Studies have yielded conflicting results regarding how modifying lipid A with pEtN alters bacterial virulence. In this study, we used in vitro and in vivo models to understand the role of *mcr* genes in bacterial virulence. Strains overexpressing *mcr* (except *mcr-2*) showed increased virulence compared with that of the control group. Our results were inconsistent with those of Yang et al., who reported that acquisition of naturally generated *mcr-1* carried by wild-type plasmids in *E. coli* markedly depleted its virulence [1]. However, in their study, acquisition of the *mcr-1*-carrying plasmid did not affect bacterial growth, and the fitness cost was moderate. Thus, we inferred that the increased virulence observed in our research might be related to fitness costs generated by MCR overexpression. MCR-mediated modification of lipid A is reported to cause profound changes in the bacterial outer membrane architecture in *E. coli* [1]; subsequently, membrane modification may further result in important membrane component release and could increase bacterial virulence in vivo [29,30]. To test our hypothesis, we detected the LPS concentrations in *G. mellonella* after bacterial infection. *G. mellonella* infected with MCR-expressing strains showed higher tissue LPS concentrations than did the control group. The result supports our hypothesis that MCR overexpression mediated lipid A modification result in LPS release and increase bacterial virulence. Our results are similar with those of Kaito Chikara et al.’s report, where, by developing a silkworm infection model, the researchers found that non-pathogenic *E. coli* could acquire virulence by mutating essential gene in growth or LPS transport, and this mutation could increase the amount of LPS in the outer membrane vesicle fraction [31]. In our trans-well assay, we observed an increased amount of bacteria expressing MCR penetrate the in vitro epithelial barrier, as it was reported that lipid A remodeling could trigger LPS liberty and the liberated LPS could increase intestinal tight junction permeability [32,33]; thus, we hypothesized that MCR expressing may help bacteria destroy epithelial barrier function by remodeling bacteria membrane and liberating the LPS. Regarding virulence and fitness costs of MCR-expressing strains, we also noticed that strains with greater fitness burden tended to be more virulent; however, depending on the present study, we could not directly correlate the variation in bacterial virulence to the fitness costs. Hence, the mechanisms of the variations in *mcr*-mediated bacterial virulence are complex and remain unclear, and therefore further studies are needed.

Our study had some limitations. We observed phenotypic differences in fitness costs and virulence among *mcr-1~5*-carrying bacteria. However, we could not explain the reason for these differences. Hence, we hypothesized some potential reasons for these differences. First, the expressions of different MCRs are precisely regulated in host bacteria, and the transcriptional level of the plasmids that carry *mcr* is controlled by both the promoter and the genomic background of the host bacteria. This is because the *mcr-1* carried by different plasmids and transformed in the same host strain (EC600) have shown the same expression at baseline [34]. Second, the previous reports confirmed different enzymatic efficiencies of different MCR proteins [35].

## 5. Conclusions

Collectively, we explored the effect of MCR-1~5 expression on host bacteria. Among MCR-1~5, MCR-1-expressing strains showed the best survival under colistin exposure and the moderate fitness costs, which might be a critical factor for the increased global dissemination of *mcr-1*. We also detected the varied effect of *mcr* genes on enhancing bacterial virulence, which may be a potential risk of increased infection along with compromising colistin efficacy. Thus, we should emphasize the importance of the *mcr* genes’ threat to human and animal health, and more attention should be paid to deciphering prevalence and mechanism of *mcr*.

## Figures and Tables

**Table 1:** Bacteria and cells used in this study.

Name	Description	Source or Reference
**Strains**		
SHP45	WT *E. coli* strain with *mcr-1* of pig origin	[17]
DH5α-*mcr-2*	Constructed *E. coli* strain with *mcr-2*	[18]
WJ1	WT *E. coli* strain with *mcr-3* of pig origin	[19,20]
D32	WT *E. coli* strain with *mcr-4* of pig origin	Laboratory collection
D13	WT *E. coli* strain with *mcr-5* of pig origin	Laboratory collection
*mcr-1*/pBAD	*E. coli* TOP10 with *mcr-1/*pBAD	This study
*mcr-2*/pBAD	*E. coli* TOP10 with *mcr-2/*pBAD	This study
*mcr-3*/pBAD	*E. coli* TOP10 with *mcr-3/*pBAD	This study
*mcr-4*/pBAD	*E. coli* TOP10 with *mcr-4/*pBAD	This study
*mcr-5*/pBAD	*E. coli* TOP10 with *mcr-5/*pBAD	This study
pBAD	*E. coli* TOP10 with pBAD	This study
ATCC25922	*E. coli*, quality control strain	Laboratory collection
**Cells**		
Caco-2	Human colon carcinoma cell line	Laboratory collection

## Data Availability

The data presented in this study are available on request from the corresponding author.

## Appendix A

**Table A1:** Primers used in this research.

Primers	Sequences	Source
*mcr*-1-F	ggctaacaggaggaattaaccatggATGATGCAGCATACTTCTGTGTG	This study
*mcr*-1-R	gccaaaacagccaagcttcgaattcTCAGCGGATGAATGCGGTG	This study
*mcr*-2-F	ggctaacaggaggaattaaccatggATGACATCACATCACTCTTG	This study
*mcr*-2-R	gccaaaacagccaagcttcgaattcTTACTGGATAAATGCCGC	This study
*mcr*-3-F	ggctaacaggaggaattaaccatggATGCCTTCCCTTATAAAAATAAAAATTG	This study
*mcr-3*-R	gccaaaacagccaagcttcgaattcTTATTGAACATTACGACATTGAC	This study
*mcr-4*-F	ggctaacaggaggaattaaccatggGTGATTTCTAGATTTAAGACGTTATC	This study
*mcr-4*-R	gccaaaacagccaagcttcgaattcCTAATACCTGCAAGGTGC	This study
*mcr-5*-F	ggctaacaggaggaattaaccatggATGCGGTTGTCTGCATTTATC	This study
*mcr-5*-R	gccaaaacagccaagcttcgaattcTCATTGTGGTTGTCCTTTTC	This study
